# Type II Odontoid Fracture: A Case Report on Intraoperative Complication and Management

**DOI:** 10.7759/cureus.70902

**Published:** 2024-10-05

**Authors:** Merih C Yilmaz, Önder Taşkın, Salih Yilmaz

**Affiliations:** 1 Neurosurgery, VM Medical Park Hospital, Samsun, TUR; 2 Neurosurgery, Amasya Kolmed Hospital, Amasya, TUR

**Keywords:** cervical, collar, fracture, fusion, odontoid

## Abstract

Type II odontoid fractures are recognized as unstable fractures, often necessitating surgical intervention. The anterior transodontoid screw technique emerges as a commonly employed surgical approach in such cases, with factors like age, osteoporosis, and the extent of fracture line displacement influencing surgical success. We report this case with the aim of highlighting the postoperative outcomes of a patient with a Type II odontoid fracture, focusing on the impact of an intraoperative complication resulting in an increased distance between the fracture lines. Our report sheds light on the management challenges posed by Type II odontoid fractures, particularly when complicated by intraoperative factors affecting fracture line alignment. This finding suggests that, in patients with favorable age and bone quality, monitoring rather than revision surgery might be appropriate.

## Introduction

Cervical fractures consist of 20% C2 vertebral fractures, with half of these being odontoid fractures [1-3]. In the elderly patient group, these fractures frequently result from falls at the same level. Conversely, in younger patients, they are commonly associated with motor vehicle accidents, sports injuries, falls from a height, and blunt head traumas [4]. The classification of odontoid fractures was introduced by Anderson and D'Alonzo in 1974 and remains in widespread use today [5].

Type I involves an oblique fracture of the superior part of the odontoid process, coinciding with the rupture of the alar ligament. Type II is identified by a transverse fracture line at the junction of the odontoid and C2 corpus. Type III fractures extend into the C2 corpus. For Type II odontoid fractures, surgical treatment is often advocated due to concerns over the rate of spontaneous fusion being reduced because of the disrupted nutrition along the fracture line and instability. Anterior odontoid screw fixation and posterior C1-C2 segmental instrumentation techniques are commonly employed options. The anterior odontoid screw technique is favored because it directly stabilizes the dens and vertebral corpus without impeding the physiological movement of the upper cervical spine [6].

## Case presentation

**Figure 1:**
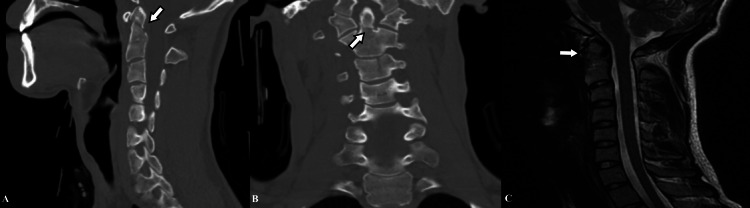
Preoperative cervical CT (A, B) and cervical sagittal MRI (C) images of the patient; fracture line is indicated by a white arrow

A 21-year-old male, in a car accident, presented to the emergency department complaining of neck pain. Neurologically, he was found to be intact. Computerized cervical tomography revealed a Type II odontoid fracture. Given his impending surgery, MRI images were procured to assess the integrity of the transverse and alar ligaments (Figure 1).

**Figure 2:**
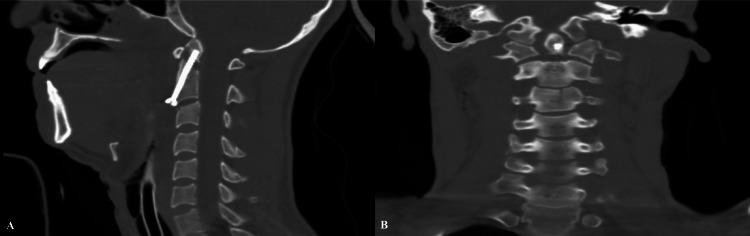
Postoperative computerized cervical tomography (A, B) showing the widened fracture gap

Before the procedure, the patient was informed about the surgical intervention and provided consent. During the surgery, after making a transverse incision at the anterior C3-4 level and accessing the paravertebral region, the C2-3 disc level was identified and retractors were placed. A K-wire was carefully guided along the midline at the inferior and anterior margin of the C2 vertebra with biplanar fluoroscopic guidance. As attempts were made to navigate the K-wire around the fracture using a high-speed drill, the broken dens segment remained elusive due to a drill malfunction. Consequently, a threaded tap screw was used in the K-wire's path to fashion the odontoid screw trajectory. Once the tapping was complete, it became evident that the fractured dens segment had dislodged from the C2 corpus. The procedure was concluded with the insertion of an odontoid screw. Early postoperative computerized tomography illustrated that while the odontoid Lag screw secured the fractured dens segment, a 7.5 mm gap persisted between the fracture components (Figure 2).

**Figure 3:**
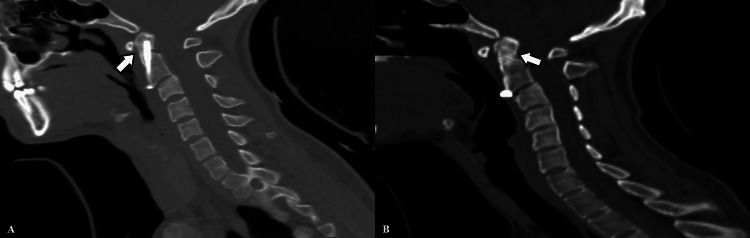
Showing anterior fusion at two months postoperatively (A). Illustrating complete fusion at the three-month follow-up on sagittal CT section (B). The fusion area is marked by white arrows

Given the patient's age, absence of comorbidities, and robust bone quality, revision surgery was not pursued. Instead, management with a Philadelphia collar was chosen. During follow-up, the patient remained neurologically intact without new complaints. Two-month postoperative imaging indicated anterior fusion progression. By the end of the third month, the patient reported no pain and was able to resume daily activities comfortably. Complete anterior-to-posterior fusion was observed by this time (Figure 3).

## Discussion

In a study involving patients aged over 60, posterior C1-2 arthrodesis fixation demonstrated higher fusion rates in developed countries when compared to anterior odontoid screw fixation. Generally, there is no substantial difference between these two surgical methods [7]. However, the posterior fusion approach may adversely affect the fusion of odontoid fractures due to an increase in sagittal balance within the cervical spine. Additionally, bone fusion at sites other than the primary fracture location can lead to delayed or unsuccessful fusions [8].

A notable advantage of the anterior odontoid screw technique is that it does not impair cervical motion, unlike posterior fusion approaches. Furthermore, research indicates that for patients under 60, employing a non-fusion technique for primary C1-C2 fixation, along with the removal of instrumentation post-odontoid fusion, can significantly enhance cervical motion function [9].

The anterior transodontoid screw technique remains a primary choice for treating Type II odontoid fractures due to its superior fusion rates, lower morbidity, and mortality, compared to conservative approaches [10]. In late-presenting cases, strategies exist that use K-wire to perforate multiple pathways on the C2 corpus and dens. This disrupts the sclerotic tissue, potentially enhancing the fusion rate, especially as sclerotic tissue on the fracture line can impede fusion [11].

Fusion rates are notably compromised when post-surgical gaps exceed 6 mm. For instance, Apuzzo et al. identified a %33 non-union rate in patients managed with external immobilization where the fracture gap was 4 mm [12]. Similarly, Hadley et al. noted a staggering 67% non-union rate when the gap reached or surpassed 6 mm [10]. Age is undeniably a crucial determinant in fusion outcomes, with those over 40 showing diminished rates [13-17].

## Conclusions

Despite a 7.5 mm postoperative fracture gap, our patient demonstrated successful fusion, which can likely be attributed to his age and excellent bone health. While using a rigid collar may be an option for certain patients, further comprehensive studies are necessary, particularly when postoperative gaps exceed 6 mm. 
